# A Comprehensive Investigation on Common Polymorphisms in the *MDR1/ABCB1* Transporter Gene and Susceptibility to Colorectal Cancer

**DOI:** 10.1371/journal.pone.0032784

**Published:** 2012-03-02

**Authors:** Daniele Campa, Juan Sainz, Barbara Pardini, Ludmila Vodickova, Alessio Naccarati, Anja Rudolph, Jan Novotny, Asta Försti, Stephan Buch, Witigo von Schönfels, Clemens Schafmayer, Henry Völzke, Michael Hoffmeister, Bernd Frank, Roberto Barale, Kari Hemminki, Jochen Hampe, Jenny Chang-Claude, Hermann Brenner, Pavel Vodicka, Federico Canzian

**Affiliations:** 1 Genomic Epidemiology Group, German Cancer Research Center (DKFZ), Heidelberg, Germany; 2 Department of Biology, University of Pisa, Pisa, Italy; 3 Division of Molecular Genetic Epidemiology, German Cancer Research Center (DKFZ), Heidelberg, Germany; 4 Institute of Experimental Medicine, Academy of Sciences of the Czech Republic, Prague, Czech Republic; 5 Division of Cancer Epidemiology, German Cancer Research Center (DKFZ), Heidelberg, Germany; 6 Department of Oncology, First Faculty of Medicine, Charles University, Prague, Czech Republic; 7 Center for Primary Health Care Research, Clinical Research Center, SUS Malmö, Malmö, Sweden; 8 Department of General Internal Medicine, University Hospital Schleswig-Holstein, Kiel, Germany; 9 POPGEN Biobank Project, University Hospital Schleswig-Holstein, Kiel, Germany; 10 Department of General and Thoracic Surgery, University Hospital Schleswig-Holstein, Kiel, Germany; 11 Institute for Community Medicine, University Hospital of the Ernst Moritz Arndt University, Greifswald, Greifswald, Germany; 12 Division of Clinical Epidemiology and Aging Research, German Cancer Research Center (DKFZ), Heidelberg, Germany; 13 Institute of Biology and Medical Genetics, First Faculty of Medicine, Charles University, Prague, Czech Republic; University of Texas School of Public Health, United States of America

## Abstract

ATP Binding Cassette B1 (*ABCB1*) is a transporter with a broad substrate specificity involved in the elimination of several carcinogens from the gut. Several polymorphic variants within the *ABCB1* gene have been reported as modulators of *ABCB1*-mediated transport. We investigated the impact of *ABCB1* genetic variants on colorectal cancer (CRC) risk. A hybrid tagging/functional approach was performed to select 28 single nucleotide polymorphisms (SNPs) that were genotyped in 1,321 Czech subjects, 699 CRC cases and 622 controls. In addition, six potentially functional SNPs were genotyped in 3,662 German subjects, 1,809 cases and 1,853 controls from the DACHS study. We found that three functional SNPs (rs1202168, rs1045642 and rs868755) were associated with CRC risk in the German population. Carriers of the rs1202168_T and rs868755_T alleles had an increased risk for CRC (P*_trend_* = 0.016 and 0.029, respectively), while individuals bearing the rs1045642_C allele showed a decreased risk of CRC (P*_trend_* = 0.022). We sought to replicate the most significant results in an independent case-control study of 3,803 subjects, 2,169 cases and 1,634 controls carried out in the North of Germany. None of the SNPs tested were significantly associated with CRC risk in the replication study. In conclusion, in this study of about 8,800 individuals we show that *ABCB1* gene polymorphisms play at best a minor role in the susceptibility to CRC.

## Introduction

Colorectal cancer (CRC) is one of the most common cancers diagnosed in Western countries [1], [2] and its etiology involves the interaction of genetic and environmental factors [3], [4], [5], [6], [7], [8], [9], [10], [11]. Although the biological processes connecting lifestyle characteristics and colorectal carcinogenesis remain unclear, association studies analyzing the influence of dietary factors have evidenced that, at least in part, CRC arises as a consequence of exposure to xenobiotics taken up with food [6], [12].

ATP-binding cassette (ABC) is one of the largest families of active efflux transporters that are located mainly in tissues acting as a barrier or having an excretory function. ABC transporters are highly expressed in the apical membrane of enterocytes where they mediate the efflux of a wide variety of endogenous substrates (including sugars, amino acids, nucleotides, steroids, inorganic ions) into the gut lumen. In addition, most of the ABC transporters play a role in cell defense against environmental attacks generated by xenobiotics [13].

*ABCB1* expression in the human intestine increases from proximal to distal, resulting in the highest expression levels in the colon [14], where it is involved in the excretion of several carcinogens from the gut into the intestinal lumen [15]. In addition, it has been demonstrated that *ABCB1* is responsible for the efflux of cooked-food mutagens [16], several tobacco-related carcinogens [17], [18], [19], as well as a broad spectrum of drugs [20], [21], [22], [23].

The expression and activity of the *ABCB1* transporter may differ between individuals, due to either genetic polymorphisms or pathological conditions [24] and, as a result, this might be reflected in differences in the bioavailability of different toxins, carcinogens and drugs [5]. Over the last decades, this hypothesis has led to a proliferation of *in vitro* reports examining the functionality of *ABCB1* gene polymorphisms and the investigation on their potential role in many diseases and cancers [4], [25], [26], [27], [28], [29], [30], [31]. Functional polymorphic variants of *ABCB1* have been reported in the Caucasian population. For example Hoffmeyer and collaborators reported that the C3435T variant (rs1045642) results in a reduced activity and expression of the protein in the duodenum [23]. Likewise, several studies have also focused on the association between polymorphic variants of ABC multidrug transporters and exposure to anticancer drugs [21], [22]. Despite numerous association studies addressing the effect of *ABCB1* variants and CRC [30], [31], [32], [33], [34], limited data are available, as most of these association studies have analyzed a reduced number of genetic markers or have been performed in a relatively small population.

The aim of the present work was to ascertain if genetic variants within the *ABCB1* gene influence the risk of CRC, with particular attention to the role of putatively functional variants that have previously been shown to be associated with cancer risk [24], [30], [32], [33].

## Materials and Methods

### Ethics statement

All participants signed an informed written consent. The study was approved by the ethical review boards of the institutions responsible for subject recruitment in each of the recruitment centres. The ethical committees were the following: Ethics Committee of the Institute of Experimental Medicine, Prague, Czech Republic. The DACHS study was approved by the ethics committees of the Medical Faculty at the University of Heidelberg and of the Medical Chambers of Baden-Württemberg and Rhineland-Palatinate. The North German Study (Kiel) was approved by the ethics committees of the Medical Faculty at the Christian-Albrechts University of Kiel and Mecklenburg- West Pomerania (Study of Health In Pomerania).

### Study populations

In this study three populations of Caucasian origin (one from the Czech Republic and two from Germany) were investigated, including a total of 8786 subjects. All the populations were previously described in detail [35]. Briefly, the first case-control study comprises 1321 subjects, 699 CRC cases and 622 controls from the Czech Republic [36]. All participants were recruited from six oncological and five gastroenterological departments, all over the Czech Republic. The study was based on incident cases with positive colonoscopic results for malignancy and with histologically confirmed colon or rectal carcinoma. Controls were defined as subjects who had undergone colonoscopy and whose colonoscopic results were negative for malignancy or colorectal adenomas. They were sampled in the same time frame as the cases. All participants signed an informed written consent and the design of the study was approved by the Ethical Committee of the Institute of Experimental Medicine, Prague, Czech Republic.

The second case-control study comprises 3662 participants, 1809 cases and 1853 controls recruited from the DACHS (Darmkrebs: Chancen der Verhütung durch Screening) study, [37]. Cases had incident invasive CRC diagnosed between January 2003 and March 2007. They were recruited from patients who received in-patient treatment in a hospital of the Rhein-Neckar-Odenwald region (Southwest Germany) due to a first diagnosis of CRC. Controls were frequency matched according to gender, county of residence and age and randomly selected from lists of population registries. The DACHS study was approved by the ethics committees of the Medical Faculty at the University of Heidelberg and of the Medical Chambers of Baden-Württemberg and Rhineland-Palatinate. All patients gave their informed written consent to participate in this study.

In order to replicate the positive findings in the discovery set, we also used an independent population-based case-control study in a North German population that comprises 3803 subjects, 2169 cases and 1634 controls [38]. CRC cases were identified through the regional cancer registry of Schleswig-Holstein or through records of surgical departments in Northern Germany and recruited by the POPGEN biobank. Controls were randomly selected from population registries and also recruited by the POPGEN biobank. Additional controls were recruited from SHIP (Study of Health In Pomerania) in North-eastern Germany and were cancer-free at recruitment.

### SNP Selection

The entire set of common genetic variants in *ABCB1* was assessed following both tagging and functional approaches. The aim of the SNP tagging was to identify a set of SNPs that efficiently captures all the known common genetic variability, while the functional approach was used to determine the impact of potentially functional variants within *ABCB1* gene on CRC risk. The tagging was carried out using the algorithm described by Carlson and co-workers [39]. All SNPs within the region of *ABCB1* (including 5 kb upstream of the first exon and downstream of the last exon) and with a minor allele frequency (MAF) ≥5% in Caucasians (International HapMap Project, version 21a; http://www.hapmap.org), were included. Tagging SNPs were selected with the use of the Haploview Tagger program (http://www.broad.mit.edu/mpg/haploview/; http://www.broad.mit.edu/mpg/tagger/) [40], [41], using pairwise tagging with a minimum r^2^ of 0.8. This resulted in a selection of 28 tagging SNPs, with a mean r^2^ of the selected SNPs with the SNPs they tag of 0.976, meaning that our selection captures to a very high degree the known common variability in this gene. Considering that the genomic region of *ABCB1* is characterized by high levels of linkage disequilibrium (LD), we postulate that such SNPs are also likely to tag any hitherto unidentified common SNPs in the gene.

In order to test the hypothesis suggesting that functional SNPs rather than tagging SNPs might affect the ABCB1-mediated efflux of DNA damaging substances, we selected six potentially functional SNPs out of the 28 previously selected (rs2229109, rs1202168, rs1045642, rs9282564, rs2214102 and rs868755), and we genotyped them in the Czech and in the German DACHS populations. The selection was based on previously published results that pointed out either the functionality of *ABCB1* variants [23], [42] or their association with various diseases [43], [44], [45].

In order to exhaustively cover all the genetic variability in the *ABCB1* locus we have checked the variants described in the 1000 genome project and we found out that 180 SNPs have not been genotyped in in HapMap. We did not include these variants since in Caucasians the frequency of the minor allele was either missing or lower than the threshold of 0.05. Searching the database of genomic variants (http://projects.tcag.ca/variation/), which is a catalogue of structural variation in the human genome, we found that in Caucasian there is only a rare (MAF = 0.03%) copy number variant (CNV) and therefore we did not attempt to genotype it. Supplementary figure S1 shows in detail the number of SNPs and subjects genotyped in each phase. Supplementary figure S2 shows the LD plot of the *ABCB1* gene, all the SNPs at the locus and the SNPs genotyped, while Supplementary table S1 shows the tagging SNPs selected in the study and the SNPs they tag.

### DNA extraction and genotyping

For the Czech subjects DNA was extracted from blood using standard proteinase K digestion followed by phenol/chloroform extraction and ethanol precipitation. German DNA samples were isolated from peripheral blood mononuclear cells or mouthwash using Flexigene Kit 250 (Qiagen, Valencia, CA, USA) and Qiagen Mini Kit (Qiagen, Valencia, CA, USA), respectively. The order of DNAs from cases and controls was randomized on PCR plates in order to ensure that an equal number of cases and controls could be analyzed simultaneously. All the genotyping was carried out using Taqman (Applied Biosystems, Foster City, CA, USA) or KASPar (KBiosciences, Hoddesdon, Hertfordshire, UK) according to the manufacturers' protocols. Repeated quality control genotypes (8% of the total) showed an average concordance of 99.9%. More details on the genotyping methods can be found elsewhere [36], [46], [47]. PCR plates were read on an ABI PRISM 7900HT instrument (Applied Biosystems). The genotypes of SNPs rs2229109, rs1202168, rs1045642, rs9282564, rs2214102 partially overlap with those used in another project for a subset of the DACHS subjects reported here [48]. The results showed in the present paper have not been previously reported.

### Statistical Analysis

Genotype distribution was examined in the cases and control populations. Hardy-Weinberg equilibrium was tested in the controls by chi square test and at the α = 1% level. We used logistic regression for multivariate analyses to assess the main effects of the genetic polymorphism on CRC risk using a log-additive inheritance model. The most common genotype in the controls was assigned as the reference category. All analyses were adjusted for age and gender. Additionally, we performed a logistic regression stratifying for the cancer site (colon *versus* rectum). All statistical analyses were performed using SAS 9.2.

Since polymorphisms selected as tagging SNPs had a low level of residual linkage disequilibrium (LD), we assumed that haplotypes were adequately captured by our tagging SNP approach, and we did not attempt a haplotype analysis in the Czech population. However we performed it for the 6 SNPs selected as functional ones. Haplotype blocks were constructed from the control genotyping data using SNP-tool http://www.dkfz.de/de/molgen_epidemiology/tools/SNPtool.html, [49] and the algorithm implemented in Haploview based on confidence bounds by Gabriel and collegues [50]. The following cut-off values were used: MAF>5%, HWE p≥0.01 and 75% of non-missing genotypes. Maximum likelihood estimates of the haplotype frequencies were generated with an expectation-maximization based algorithm implemented in the PROC HAPLOTYPE procedure of SAS. Unconditional logistic regression adjusted for age (continuous), sex and study centre was used to calculate risk estimates. The most frequent haplotype was set as the reference, whereas haplotypes with a frequency below 0.05 were declared as rare haplotypes and combined.

To evaluate the possibility of false positives, we took into account the issue of multiple testing. SNPSpD software (http://genepi.qimr.edu.au/general/daleN/SNPSpD), based on the use of the spectral decomposition (SpD) of matrices of pairwise LD between SNPs, was used to calculate the effective number of independent markers (M_eff_) for multiple testing [51], [52]. The p-values were evaluated in light of the M_eff_ value.

## Results

We performed a case-control study using three nested sets of *ABCB1* SNPs in three distinct populations of Czech and German origins. The first SNP set consisted of 28 tagging SNPs, which we tested in 699 cases and 622 controls from the Czech Republic. The second SNP set consisted of a subgroup of the 28 tagging SNPs, namely six putatively functional SNPs, which we additionally typed in a German population of 1809 cases and 1853 controls. Finally, we replicated the best hits, namely SNPs rs1202168, rs1045642, rs9282564, and rs868755, in up to 2169 additional cases and 1634 additional controls from North Germany. These four SNPs were therefore typed in all the populations, giving this study a final sample size of up to 4677 cases and 4109 controls. The relevant characteristics of the three populations are reported in table 1. The genotype frequencies among the controls were in Hardy-Weinberg equilibrium for all the SNPs and in all populations. The calculated M_eff_ value was 16. We therefore considered a study-wide significance p-value threshold of 0.05/16 = 0.003.

**Table 1:** Population characteristics.

**Czech population**		Cases	Controls
		(*n* = 699)	(*n* = 622)
Males (%)*		397 (56.8)	336 (54.0)
Females (%)		302 (43.2)	286 (46.0)
Age (Age at Diagnosis)	Median	62	56
	Range	27–90	28–91
	Mean (SD)	61.1 (11.0)	55.3 (13.5)
Rectal cancer (%)		38.7	
Colon cancer (%)		61.3	
**DACHS population**		Cases	Controls
		(*n* = 1809)	(*n* = 1853)
Males (%)*		1056 (58.4)	1095 (59.1)
Females (%)		753 (41.6)	758 (40.9)
Age (Age at Diagnosis)	Median	69	70
	Range	33–94	34–98
	Mean (SD)	68.3 (10.4)	68.6 (10.2)
Rectal cancer (%)		39.0	
Colon cancer (%)		61.0	
**Kiel population**		Cases	Control
		(*n* = 2169)	(*n* = 2060)
Males (%)*		1087 (50.1)	986 (47.9)
Females (%)		1082 (49.9)	1074 (52.1)
Age (Age at Diagnosis)	Median	62	65
	Range	10–85	23–81
	Mean (SD)	60.9 (8.8)	64.7 (10.0)
Rectal cancer (%)		51.4	
Colon cancer (%)		48.6	

### Results in the discovery set

The analysis of the 28 tagging SNPs in the Czech population did not reveal any statistically significant association (using the threshold of p = 0.003). The distribution of the genotypes of the 28 SNPs in the Czech population and their odds ratios (ORs) for association with CRC risk are shown in Supplementary Table S2.

Associations of three polymorphisms with CRC risk at the conventional level of p<0.05 were observed in the DACHS study. Carriers of the rs1202168_T and rs868755_T alleles had an increased risk of CRC (OR_het_ = 1.20, 95%CI 1.04–1.40, OR_hom_ = 1.23, 95%CI 1.01–1.48, P*_trend_* = 0.016, and OR_het_ = 1.17, 95%CI 1.00–1.36, OR_hom_ = 1.22, 95%CI 1.01–1.48, P*_trend_* = 0.029, respectively) while individuals bearing the rs1045642_C allele had a decreased risk of CRC (OR_hom_ = 0.79, 95%CI 0.66–0.96, P*_trend_* = 0.022, Supplementary Table S3).

### Results of the replication study

Four SNPs which showed a statistically significant (rs1202168, rs1045642, rs868755) or borderline significant (rs9282564) association in the German discovery set were typed in an additional set of CRC cases and healthy controls from Germany. None of the SNPs showed a statistically significant association with CRC risk in the replication set or in the combined dataset. The distribution of the genotypes of the four SNPs typed in all the subjects of this study and their ORs for association with CRC risk are shown in Table 2.

**Table 2:** Replication of *ABCB1* polymorphisms.

	CZECH^a^ population		DACHS^a^ population		KIEL^a^ population		Total^a^ population		Czech population		DACHS population		KIEL population		Total population	
	Controls	Cases	Controls	Cases	Controls	Cases	Controls	Cases	OR (95% CI)^b^	*p* trend	OR (95% CI)^b^	*p* trend	OR (95% CI)^b^	*p* trend	OR (95% CI)^b^	*p* trend
***rs868755***																
G/G	201	215	568	498	346	239	1115	952	1.00	0.884	1.00	0.029	1.00	0.443	1.00	0.086
G/T	227	295	794	805	521	335	1542	1435	1.22 (0.94–1.60)		1.17 (1.00–1.36)		0.81 (0.63–1.05)		1.11 (0.99–1.25)	
T/T	92	92	344	364	162	115	598	571	0.93 (0.65–1.34)		1.22 (1.01–1.48)		0.94 (0.67–1.32)		1.12 (0.97–1.29)	
***rs1202168***																
C/C	206	231	647	568	551	726	1404	1525	1.00	0.912	1.00	0.016	1.00	0.614	1.00	0.263
C/T	274	329	835	880	795	1001	1904	2210	1.11 (0.86–1.43)		1.20 (1.04–1.40)		0.96 (0.83–1.11)		1.07 (0.98–1.18)	
T/T	112	118	318	341	284	357	714	816	0.94 (0.67–1.31)		1.23 (1.01–1.48)		0.96 (0.79–1.16)		1.06 (0.93–1.20)	
***rs1045642***																
T/T	166	190	486	501	313	192	965	883	1.00	0.797	1.00	0.022	1.00	0.051	1.00	0.297
T/C	279	335	868	918	510	354	1657	1607	1.04 (0.80–1.37)		1.03 (0.88–1.20)		1.14 (0.87–1.49)		1.06 (0.95–1.19)	
C/C	131	142	447	367	202	150	780	659	0.95 (0.68–1.32)		0.79 (0.66–0.96)		1.39 (1.00–1.92)		0.92 (0.80–1.05)	
***rs9282564***																
A/A	432	464	1471	1417	1004	1355	2907	3236	1.00	0.709	1.00	0.052	1.00	0.719	1.00	0.123
A/G	129	136	301	341	245	369	675	846	1.03 (0.78–1.37)		1.18 (1.00–1.41)		1.12 (0.93–1.34)		1.12 (1.00–1.26)	
G/G	10	12	21	24	18	13	49	49	1.20 (0.51–2.83)		1.20 (0.67–2.17)		0.53 (0.26–1.09)		0.92 (0.62–1.38)	

a Numbers may not add up to 100% of subjects due to genotyping failure.

b Models were adjusted for age, sex and (in the combined population) study.

### Haplotype analysis

None of the haplotypes showed a statistically significant association after correction for multiple testing. When testing the haplotypes in the pooled population, none showed any statistically significant association with CRC risk at the conventional threshold of P = 0.05. The distribution of *ABCB1* haplotypes for the functional SNPs and risk of CRC are shown in supplementary table S4.

## Discussion

Although functional data regarding *ABCB1* SNPs are not totally consistent, some of them have reported to be involved with an enhanced efflux transporting ability [23], [42], [53]. Likewise, several of these functional polymorphisms have been found to be associated with CRC risk factors such as ulcerative colitis or obesity [54], [55], [56]. The present study attempted to evaluate systematically the role of common *ABCB1* polymorphisms on CRC risk.

Several studies have investigated the possible role of genetic variants in *ABCB1* and risk of colorectal disease, mostly focusing on C3435T (rs1045642) and on 2677G>T/A (rs2032582). Overall these studies are heterogeneous in terms of size, number of SNPs tested, methodology used and ethnicity. C3435T was found to be associated with disease risk in several case-control studies [33], [57], [58], [59] while others did not find any statistically significant associations [34], [60] The polymorphism variant 2677G>T/A (rs2032582) in exon 21 has also been found associated with CRC risk [26], [45], [59], although not in all studies [34]. Anderson and colleagues also tested the possible association of rs3789243 in two distinct studies. They found an association in a Danish population but did not find anything in a study based on Norwegian subjects [3], [57]. In this study we have genotyped rs3789243 and we did not observe any statistically significant association.

Three *ABCB1* variants (rs1202168, rs1045642 and rs868755) were found to be associated with CRC risk in the DACHS study population. Carriers of the rs1202168_T and rs868755_T alleles showed an increased risk of CRC, while individuals bearing the, rs1045642_C allele (also referred to as C3435 in previous studies) showed a decreased risk for CRC. The results regarding rs1202168 and rs868755 are novel, although rs2032582 and rs868755 are in high LD (r^2^ = 0.85), while the results regarding rs1045642 are in concordance with several other studies that found that the C3435T polymorphism contribute to risk of CRC. In agreement with our results, Kurzawski and co-workers found that carriers of the variant allele of the C3435T polymorphism had an increased risk of CRC while carriers of CC genotype had the lowest risk for CRC [33]. In addition, several haplotypes containing the rs1045642_C allele have been associated with slower CRC progression [30]. Kimchi-Sarfaty and co-workers also suggested that this silent polymorphism affect the post-translational protein folding and its transport activities [61].

However, current data regarding the influence of *ABCB1* SNPs on CRC risk are conflicting and, although three SNPs in the DACHS population showed a statistically significant association with CRC, none of them showed the same trend in the replication study.

In addition, it is worth to note that our replication study performed in a Northern German population from Kiel showed that the rs1045642_C allele was associated with an increased, albeit not statistically significant, risk for CRC, i.e., an opposite effect to the one found in the DACHS study. These results obtained in the Northern Germans are in concordance with those previously published in a Danish population [57]. Consequently, it might be conceivable to think that the interplay between genetic variants and environmental factors could modify the risk of CRC.

Genome-wide association studies (GWAS) of CRC have confirmed the hypothesis that part of the heritable risk for this disease is caused by common, low-risk variants and have identified various common variants associated with CRC risk [62], [63], [64], [65], [66] but none of the GWAS reported polymorphisms in the *ABCB1* gene as major CRC risk factors. However, since only associations significant at the genome-wide level (typically p<10^−7^) are reported in GWAS it is not possible to exclude that *ABCB1* polymorphisms were associated with CRC risk but were not reported due to the strict statistical threshold used. It has been indeed documented that GWAS are prone to reporting false negative results [67].

To our knowledge, the present study is one of the largest studies to address the potential association between *ABCB1* polymorphisms and CRC risk. In this study we had a statistical power greater than 80% to detect an association for a log additive model with OR = 1.45 at α = 0.001 for the tagSNPs discovery set (MAF = 0.30), OR = 1.23 for the functional discovery set (MAF = 0.30) and OR = 1.16 for the joint analysis (MAF = 0.30). Moreover the hybrid tagging/functional approach facilitated a comprehensive analysis of common variants in the *ABCB1* gene region in two well-defined and homogeneous study populations with a sufficient size. In addition, we used a large population-based study carried out in the same ethnic group for replication. Nonetheless, this study has some potential limitations. First, the large number of test performed could lead to chance finding and indeed, adding the correction for multiple testing none of the SNPs showed statistically significant associations. Moreover, even if the three populations are of Caucasian origin, a possible heterogeneity between them could still be present. Finally in this report we did not consider rare variants either as SNPs or CNVs.

In conclusion in this report we cannot completely exclude a role, albeit minor, for *ABCB1* gene variants and CRC risk.

## Supporting Information

Figure S1 Shows in detail the number of SNPs and subjects genotyped in each phase.
(PPT)

Figure S2 LD plot of the *ABCB1* gene showing all the SNPs at the locus and the tagging selection. In the top left corner are shown the locus position and its size. The numbers in the diamonds are r^2^ values and the SNPs indicated with an * are the tagging SNPs selected for this study.
(TIF)

Table S1 Tagging SNPs selected in the study and the SNPs they tag.
(DOC)

Table S2 Distribution of *ABCB1* polymorphisms and risk of CRC in the Czech population.
(DOC)

Table S3 Distribution of *ABCB1* polymorphisms and risk of CRC in the German DACHS population.
(DOC)

Table S4 Distribution of *ABCB1* haplotypes for the functional SNPs and risk of CRC.
(XLS)
