# Supplementary material for: A Comprehensive Investigation on Common Polymorphisms in the MDR1/ABCB1 Transporter Gene and Susceptibility to Colorectal Cancer
Source: PLoS One. 2012 Mar 2;7(3):e32784. doi: 10.1371/journal.pone.0032784 (PMC3292569; doi:10.1371/journal.pone.0032784)
Supplement: Figure S1 — Shows in detail the number of SNPs and subjects genotyped in each phase. (PPT) [file pone.0032784.s001.ppt]

## Slide 1
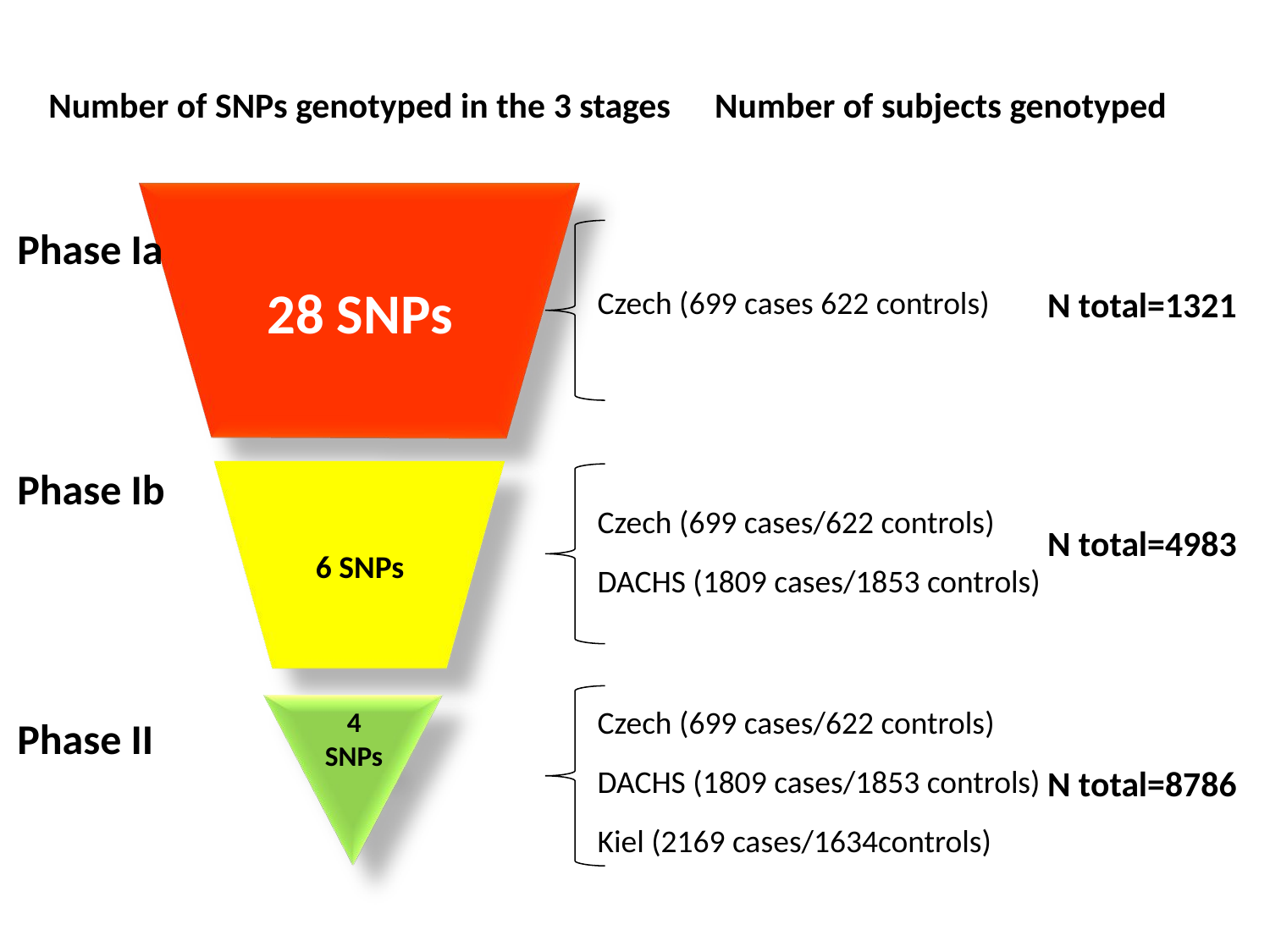

Number of SNPs genotyped in the 3 stages
Number of subjects genotyped
28 SNPs
Phase Ia
Czech (699 cases 622 controls)
N total=1321
Phase Ib
6 SNPs
Czech (699 cases/622 controls)
DACHS (1809 cases/1853 controls)
N total=4983
4 SNPs
Czech (699 cases/622 controls)
DACHS (1809 cases/1853 controls)
Kiel (2169 cases/1634controls)
Phase II
N total=8786
