# Supplementary figures and images for: A Comprehensive Investigation on Common Polymorphisms in the MDR1/ABCB1 Transporter Gene and Susceptibility to Colorectal Cancer
Source: PLoS One. 2012 Mar 2;7(3):e32784. doi: 10.1371/journal.pone.0032784 (PMC3292569; doi:10.1371/journal.pone.0032784)

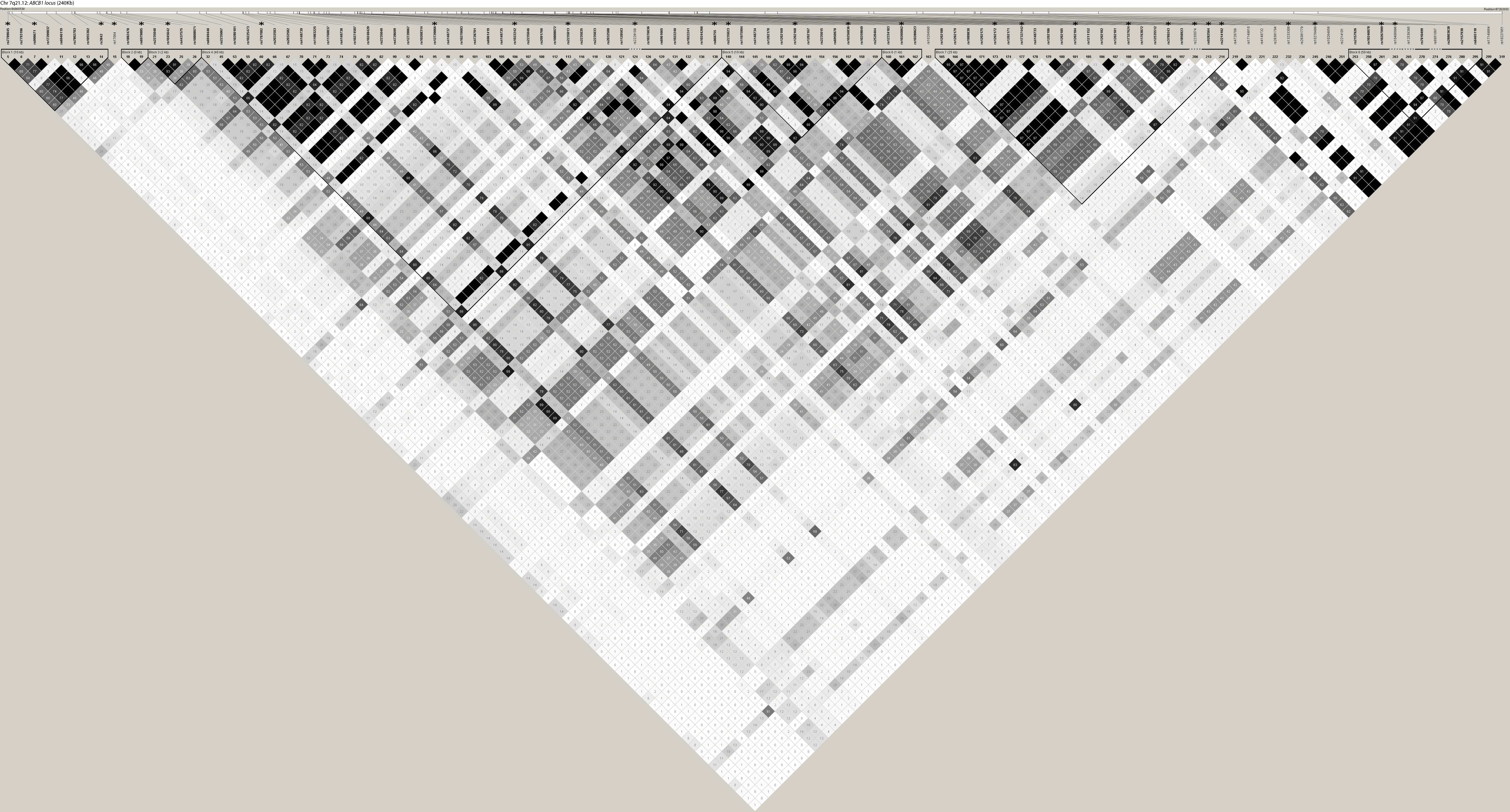

Supplement: Figure S2 — LD plot of the ABCB1 gene showing all the SNPs at the locus and the tagging selection. In the top left corner are shown the locus position and its size. The numbers in the diamonds are r2 values and the SNPs indicated with an * are the tagging SNPs selected for this study. (TIF) [file pone.0032784.s002.tif]
