# Supplementary material for: A Comprehensive Investigation on Common Polymorphisms in the MDR1/ABCB1 Transporter Gene and Susceptibility to Colorectal Cancer
Source: PLoS One. 2012 Mar 2;7(3):e32784. doi: 10.1371/journal.pone.0032784 (PMC3292569; doi:10.1371/journal.pone.0032784)
Supplement: Table S1 — Tagging SNPs selected in the study and the SNPs they tag. (DOC) [file pone.0032784.s003.doc]

**Supplementary Tables**

**Supplementary Table S1. Details of selected and genotyped SNPs in *ABCB1* gene.**

| Gene | Position | Tag SNPs | Alleles captured (r^2^>0.8) |
| --- | --- | --- | --- |
| *ABCB1* | *Chr 7q21.12* | ***rs10267099*** | rs10267099, rs2157930, rs10246878 |
|  |  | ***rs10264990*** | rs10264990 |
|  |  | ***rs7787082*** | rs10225473, rs10274587, rs2373589, rs10244266, rs10268314, rs11760837, rs11979702, rs4148740, rs10248420, rs10280101, rs7787082, rs11772987, rs12720067, rs2235040, rs2235067, rs2032583, rs1882479, rs11983225, rs10276603, rs4148739 |
|  |  | ***rs10276499*** | rs17149864, rs10264856, rs10276499, rs12539395, rs2157926, rs12540931, rs4728709, rs7790722, rs6465118, rs2157929 |
|  |  | rs10260862 | rs2235015, rs10280623, rs12334183, |
|  |  | ***rs1202172*** | rs1202179, rs1202174, rs1202185, rs1202175, rs1202182, rs1202171, rs1202181, rs1202186, rs1202180, rs1989830, rs1989831, rs1202172 |
|  |  | ***rs1202184*** | rs11763872,rs1202184 |
|  |  | ***rs2235013*** | rs1202170, rs2235033, rs2235013, rs10808072, rs6961665, rs12704364 |
|  |  | ***rs12720066*** | rs12720066 |
|  |  | ***rs13233308*** | rs13233308 |
|  |  | ***rs17064*** | rs17064 |
|  |  | ***rs3842*** | rs1055302, rs17209837, rs12672720, rs7802783, rs3842 |
|  |  | ***rs17327442*** | rs17327442, rs4148733, rs4148732 |
|  |  | ***rs17327624*** | rs17327624 |
|  |  | ***rs3789243*** | rs7802773, rs13229143, rs1858923, rs3789243, rs2188526 |
|  |  | ***rs2214102*** | rs2214102, rs1211152 |
|  |  | ***rs1045642*** | rs1002204, rs4437575, rs2235048, rs1045642 |
|  |  | ***rs2235074*** | rs2888599, rs2235074, rs10486996, rs2188525, rs2188528, rs3213619, rs2214103 |
|  |  | ***rs10256836*** | rs1922241, rs956825, rs4148734, rs10256836, rs10259849, rs1922240, rs2235035, rs6950978, rs13239201, rs13237132 |
|  |  | ***rs1922242*** | rs4148735, rs6961419, rs2091766, rs1922242, rs4148737 |
|  |  | ***rs6979885*** | rs6979885 |
|  |  | ***rs2235023*** | rs2235023, rs7810499, rs2032588 |
|  |  | ***rs868755*** | rs11975994, rs6949448, rs2235046, rs4728700, rs2520464, rs1202167, rs868755**, rs1202168**, rs4148743, rs3789244, rs4148738, rs1202169, rs10276036, rs12535512, rs10234411, rs2373586, rs1128503, rs4728702 |
|  |  | ***rs9282564*** | rs13226726, rs9282564 |
|  |  | ***rs7789645*** | rs7789645 |
|  |  | ***rs998671*** | rs998671 |
|  |  | ***rs2229109*** | rs2229109 |
