# Supplementary material for: A Comprehensive Investigation on Common Polymorphisms in the MDR1/ABCB1 Transporter Gene and Susceptibility to Colorectal Cancer
Source: PLoS One. 2012 Mar 2;7(3):e32784. doi: 10.1371/journal.pone.0032784 (PMC3292569; doi:10.1371/journal.pone.0032784)
Supplement: Table S2 — Distribution of ABCB1 polymorphisms and risk of CRC in the Czech population. (DOC) [file pone.0032784.s004.doc]

**Supplementary** **Table S2**. Distribution of *ABCB1* polymorphisms and risk of CRC in Czech population

| **SNP** | **Cases^a^** | **Controls^a^** | **OR (95% CI)^b^** | **P trend** |
| --- | --- | --- | --- | --- |
|  |  |  |  |  |
| rs10256836 |  |  |  |  |
| C/C | 287 | 255 | 1 |  |
| C/G | 294 | 257 | 0.99 (0.78-1-27) |  |
| G/G | 76 | 71 | 0.83 (0.57-1.22) | 0.88 |
| rs10267099 |  |  |  |  |
| A/A | 423 | 342 | 1 |  |
| A/G | 235 | 187 | 1.07 (0.84-1.37) |  |
| G/G | 31 | 17 | 0.99 (0.55-1.82) | 0.41 |
| rs10276499 |  |  |  |  |
| C/C | 540 | 505 | 1 |  |
| C/T | 78 | 73 | 0.99 (0.71-1.40) |  |
| T/T | 7 | 5 | 1.30 (0.41-4.15) | 0.82 |
| rs1202172 |  |  |  |  |
| T/T | 185 | 140 | 1 |  |
| T/G | 297 | 298 | 1.04 (0.81-1.32) |  |
| G/G | 170 | 148 | 0.78 (0.52-1.15) | 0.37 |
| rs17064 |  |  |  |  |
| A/A | 600 | 532 | 1 |  |
| A/T | 70 | 59 | 1.07 (0.73-1.57) |  |
| T/T | 3 | 1 | 2.55 (0.22-29.3) | 0.58 |
| rs1922242 |  |  |  |  |
| A/A | 198 | 175 | 1 |  |
| A/T | 308 | 287 | 0.96 (0.73-1.26) |  |
| T/T | 139 | 124 | 0.90 (0.64-1.25) | 0.91 |
| rs2235023 |  |  |  |  |
| G/G | 594 | 533 |  |  |
| G/A | 61 | 57 | 1.01 (0.68-1.50) |  |
| A/A | 5 | 3 | 1.31 (0.29-5.91) | 0.94 |
| rs2235013 |  |  |  |  |
| C/C | 167 | 166 | 1 |  |
| C/T | 342 | 277 | 1.29 (0.98-1.70) |  |
| T/T | 147 | 143 | 1.09 (0.79-1.51) | 0.83 |
| rs2235074 |  |  |  |  |
| T/T | 629 | 551 | 1 |  |
| T/C | 42 | 39 | 0.98 (0.62-1.56) |  |
| C/C | 4 | 1 | 2.84 (0.31-26.3) | 0.76 |
| rs3789243 |  |  |  |  |
| T/T | 182 | 144 | 1 |  |
| T/C | 317 | 275 | 0.90 (0.68-1.19) |  |
| C/C | 164 | 168 | 0.82 (0.59-1.12) | 0.09 |
| rs998671 |  |  |  |  |
| G/G | 520 | 462 | 1 |  |
| G/A | 144 | 122 | 1.04 (0.79-1.39) |  |
| A/A | 7 | 8 | 0.94 (0.31-2.83) | 0.92 |
| rs2214102 |  |  |  |  |
| G/G | 518 | 479 | 1 |  |
| G/A | 124 | 112 | 1 (0.74-1.34) |  |
| A/A | 13 | 6 | 1.93 (0.70-5.27) | 0.40 |
|  |  |  |  |  |

**Supplementary** **Table 1.** Continued

| **SNP** | **Cases^a^** | **Controls^a^** | **OR (95% CI)^b^** | **P trend** |
| --- | --- | --- | --- | --- |
|  |  |  |  |  |
| rs7787082 |  |  |  |  |
| A/A | 486 | 425 | 1 |  |
| A/G | 164 | 146 | 1.01 (0.77-1.32) |  |
| G/G | 14 | 17 | 0.75 (0.36-1.57) | 0.55 |
| rs3842 |  |  |  |  |
| G/G | 519 | 463 | 1 |  |
| G/A | 138 | 126 | 0.96 (0.72-1.28) |  |
| A/A | 10 | 10 | 1.07 (0.42-2.759 | 0.83 |
| rs1045642 |  |  |  |  |
| C/C | 190 | 166 | 1 |  |
| C/T | 335 | 279 | 1.04 (0.80 - 1.37) |  |
| T/T | 142 | 131 | 0.95 (0.68 - 1.32) | 0.80 |
| rs12233308 |  |  |  |  |
| G/G | 185 | 140 | 1 |  |
| G/A | 297 | 298 | 0.69 (0.52-0.92) |  |
| A/A | 170 | 148 | 0.86 (0.62-1.19) | 0.37 |
| rs10260862 |  |  |  |  |
| C/C | 432 | 411 | 1 |  |
| C/G | 166 | 131 | 1.18 (0.89-1.55) |  |
| G/G | 24 | 25 | 0.99 (0.54-1.80) | 0.60 |
| rs10264990 |  |  |  |  |
| T/T | 277 | 258 | 1 |  |
| C/T | 285 | 257 | 1.05 (0.82-1.35) |  |
| C/C | 94 | 73 | 1.09 (0.75-1.56) | 0.83 |
| rs1202184 |  |  |  |  |
| A/A | 147 | 159 | 1 |  |
| G/A | 340 | 279 | 1.30 (0.98-1.73) |  |
| G/G | 179 | 157 | 1.15 (0.83-1.59) | 0.20 |
| rs12720066 |  |  |  |  |
| T/T | 509 | 517 | 1 |  |
| T/G | 41 | 31 | 1.34 (0.83-2.17) |  |
| G/G | 1 | 0 | 1.01 (0.63-16.3) | 0.25 |
| rs17327442 |  |  |  |  |
| A/A | 452 | 430 | 1 |  |
| A/T | 185 | 144 | 1.22 (0.80-1.34) |  |
| T/T | 34 | 20 | 1.62 (0.83-2.72**)** | **0.03** |
| rs17327624 |  |  |  |  |
| T/T | 410 | 365 | 1 |  |
| T/G | 201 | 175 | 1.04 (0.80-1.34) |  |
| G/G | 34 | 23 | 1.26 (0.71-1.24) | 0.42 |
| rs7789645 |  |  |  |  |
| C/C | 454 | 431 | 1 |  |
| C/G | 188 | 150 | 1.15 (0.89-1.50) |  |
| G/G | 16 | 13 | 1.15 (0.52-2.54) | 0.18 |

**Supplementary** **Table 1**. Continued

| **SNP** | **Cases^a^** | **Controls^a^** | **OR (95% CI)^b^** | **P trend** |
| --- | --- | --- | --- | --- |
|  |  |  |  |  |
| rs9282564 |  |  |  |  |
| A/A | 464 | 432 | 1 |  |
| G/A | 136 | 129 | 1.03 (0.78 - 1.37) |  |
| G/G | 12 | 10 | 1.20 (0.51 - 2.83) | 0.71 |
| rs6979885 |  |  |  |  |
| A/A | 308 | 239 | 1 |  |
| A/G | 241 | 227 | 0.86 (0.67-1.11) |  |
| G/G | 59 | 36 | 1.30 (0.85-2.06) | 0.89 |
| rs2229109 |  |  |  |  |
| G/G | 616 | 546 | 1 |  |
| A/G | 58 | 56 | 0.92 (0.62 - 1.37) |  |
| A/A | 0 | 2 | NA | 0.48 |
| rs1202168 |  |  |  |  |
| C/C | 231 | 206 | 1 |  |
| C/T | 329 | 274 | 1.11 (0.86 - 1.43) |  |
| T/T | 118 | 112 | 0.94 (0.67 - 1.31) | 0.91 |
| rs868755 |  |  |  |  |
| G/G | 215 | 201 | 1 |  |
| G/T | 295 | 227 | 1.22 (0.94 - 1.60) |  |
| T/T | 92 | 92 | 0.93 (0.65 - 1.34) | 0.88 |
|  |  |  |  |  |

^a^ Numbers may not add up to 100% of subjects due to genotyping failure. All samples that did not give a reliable result in the first round of genotyping were resubmitted to up to two additional rounds of genotyping. Data points that were still not filled after this procedure were left blank.

^b^ OR: odds ratio; CI: confidence interval. Adjusted for gender and age.
