# Supplementary material for: A Comprehensive Investigation on Common Polymorphisms in the MDR1/ABCB1 Transporter Gene and Susceptibility to Colorectal Cancer
Source: PLoS One. 2012 Mar 2;7(3):e32784. doi: 10.1371/journal.pone.0032784 (PMC3292569; doi:10.1371/journal.pone.0032784)
Supplement: Table S3 — Distribution of ABCB1 polymorphisms and risk of CRC in the German DACHS population. (DOC) [file pone.0032784.s005.doc]

**Supplementary** **Table 3**. Distribution of *ABCB1* polymorphisms and risk of CRC in German DACHS population

| **SNP** | **Cases^a^** | **Controls^a^** | **OR (95% CI)^b^** | **P trend** |
| --- | --- | --- | --- | --- |
| rs2229109 |  |  |  |  |
| G/G | 1610 | 1612 | 1 |  |
| A/G | 174 | 189 | 0.92 (0.74 - 1.14) |  |
| A/A | 4 | 3 | 1.33 (0.30 - 5.96) | 0.525 |
| rs1202168 |  |  |  |  |
| C/C | 568 | 647 | 1 |  |
| T/C | 885 | 835 | 1.20 (1.04 - 1.40) |  |
| T/T | 342 | 318 | 1.23 (1.01 - 1.48) | **0.016** |
| rs1045642 |  |  |  |  |
| T/T | 504 | 486 | 1 |  |
| T/C | 921 | 868 | 1.03 (0.88 - 1.20) |  |
| C/C | 367 | 447 | 0.79 (0.66 - 0.96) | **0.022** |
| rs9282564 |  |  |  |  |
| A/A | 1421 | 1471 | 1 |  |
| A/G | 343 | 301 | 1.18 (1.00 - 1.41) |  |
| G/G | 24 | 21 | 1.20 (0.67 - 2.17) | 0.052 |
| rs2214102 |  |  |  |  |
| G/G | 1521 | 1552 | 1 |  |
| A/G | 263 | 237 | 1.13 (0.94 - 1.37) |  |
| A/A | 12 | 9 | 1.37 (0.57 - 3.25) | 0.152 |
| rs868755 |  |  |  |  |
| A/A | 568 | 499 | 1.00 |  |
| A/C | 794 | 810 | 1.17 (1.00 - 1.36) |  |
| C/C | 344 | 364 | 1.22 (1.01 - 1.48) | **0.029** |

^a^ OR: odds ratio; CI: confidence interval. Adjusted for gender and age. Statistically significant results are shown in bold.
